# Rapid Drop-Test for Lectin Binding with Glycopolymer-Coated Optical Ring Resonators

**DOI:** 10.3390/bios9010024

**Published:** 2019-02-12

**Authors:** Christine Schulte-Osseili, Moritz Kleinert, Norbert Keil, Ruben R. Rosencrantz

**Affiliations:** 1 Fraunhofer Institute for Applied Polymer Research IAP, Geiselbergstr. 69, 14476 Potsdam, Germany; christine.schulte-osseili@iap.fraunhofer.de; 2 Fraunhofer Institute for Telecommunications, Heinrich Hertz Institute, HHI, Einsteinufer 37, 10587 Berlin, Germany; moritz.kleinert@hhi.fraunhofer.de (M.K.); norbert.keil@hhi.fraunhofer.de (N.K.)

**Keywords:** optical ring resonator, glycopolymers, lectins, glycan interactions

## Abstract

We fabricated a simple sensor system for qualitative analysis of glycan-mediated interactions. Our main aim was to establish a ronbbust system that allowes drop-tests without complex fluidics. The test system should be usable in routine analytics in the future and bear sufficient sensitivity to detect binding events in the nanomolar range. For this, we employed optical ring resonators and coated them with high avidity glycopolymers based on *N*-acetylglucosamine (GlcNAc). These hydrophilic polymers are also very feasible in preventing unspecific protein adsorption. Drop-on binding studies with suitable lectins showed that glycopolymers were specifically recognized by a lectin with GlcNAc-specificity and prevented unspecific protein interactions very well. The system could be elaborated in the future for detection of glycan-mediated interactions in the biomedical field and is promising in means of multiplexed analysis and usage in routine analysis.

## 1. Introduction

Lectins are ubiquitous carbohydrate-binding proteins and gain more and more interest in research as they may serve as novel diagnostic disease markers [1,2]. Several adhesins and toxins from microbial pathogens, such as *Escherichia coli*, *Clostridium difficile* or *Pseudomonas aeruginosa,* bear a lectin domain, that is used to bind to the host cell surface and induce pathogenicity in that way [3]. Additionally, lectins play a role in tumor progression, angiogenesis and metastasis in human, so that early diagnosis and novel treatments may target lectins in the future [3,4]. Up to today, no commercial sensor system for easy analysis of lectin binding is available. Most binding studies are performed in a well-plate format, utilizing ELISA setups, or glycan arrays [5,6]. Some highly sensitive sensor platforms based on electrochemical approaches [7,8], surface plasmon resonance [9,10], or others are published but suffer so far from laborious production processes, rather complex measuring setups or do not take the tremendous amount of unspecific binding of lectins to surfaces in general into account. In the past we have employed a sensor system, based on glycopolymers as ligands, which showed excellent repellence of unspecific protein adsorption on the one hand, paired with excellent binding efficiency due to the multivalent ligand presentation for lectins on the other [11]. Therefore, the usage of glycopolymers as specific lectin biosensors is highly advantageous for these systems.

In this work we aim for a simple, multiplexable and easy to produce sensor system with sufficient sensitivity and the possibility of scalability, in means of production of large numbers of sensors. Optical ring resonators are the matter of choice here, as they bring in all requested properties. They rely on the self-interference of light, propagating through a closed waveguide loop, resulting in sharp resonances at optical wavelengths at which the resonance condition is satisfied [12]. A change of the refractive index at the ring resonator surface yields a change of the effective refractive index of the propagating optical mode and hence a shift of the resonance wavelengths. This concept allows for the realization of sensitive optical sensors for refractive index changes with sensitivities up to 200 nm/RIU [13]. By introducing a functionalization of the ring resonator, the measured resonance shift becomes proportional to the concentration of molecules that bind to the surface. Using this concept, the specific detection of a wide array of molecules, such as 1,3,5-trinitrotoluene (TNT), avidin, and C-reactive protein (CRP), was successfully demonstrated [14,15,16]. The fabrication by lithographic processes on wafer scale allows for multiplexing of several optical ring resonators, enabling the redundant measurements to increase the reproducibility or a multi-parameter measurement via different surface functionalization of individual resonators [17]. The devices in this work feature a free spectral range (FSR) of 0.6 nm between the resonances. This enables the use of low-cost distributed feedback laser diodes with a thermal wavelength tuning range of some nanometers to interrogate the spectral response of the ring resonators in future readout systems. In contrast, most work on functionalized ring resonators focused on small ring circumferences with resulting FSRs in the order of some tens of nanometers [18], which required complex and expensive laser systems for the evaluation. In addition to their small FSR, the optical ring resonators in this work operate at wavelengths in the fiber–optical band, around 1550 nm. This allows for the use of cost-effective and small standard telecommunications optoelectronic components for light delivery and detection. It hence opens up the possibility to develop a hand-held sensor system based on the approach presented here. The so far reported approaches for using optical ring resonators for detection of lectin binding involve a setup containing a fluidics, which limits on the one hand the ease of handling, but also elongates the measurement time [18]. Fluidical systems are necessary to allow the determination of kinetical parameters of the biomolecular interaction, but are as well of interest to allow washing of the sensor surface to remove nonspecifically bound protein. For an application in clinical laboratories, short measurement times, easy to handle systems like simple drop- or spot-on tests are clearly the matter of choice.

We aim for a drop-test to detect the presence or absence of lectins by utilizing their carbohydrate binding specificity. The label-free system should be multiplexable, scalable in production, simple in usage, and should provide clear yes/no answers in short times (minutes). Due to omitting any fluidical system, the sensor surface must be coated with a layer that prevents nonspecific protein interactions almost totally. The application in mind is the detection of toxins or adhesins in complex fluids for diagnosis. But first, this preliminary study investigated the suitability of optical ring resonators and glycopolymers for this task.

## 2. Materials and Methods

### 2.1. Materials

All chemicals were used as received without further purification. *N*-acetyl-d-glucosamine (GlcNAc), sodium methoxide (NaOCH_3_), acetyl chloride and silver perchlorate monohydrate (AgClO_4_∙H2O) were purchased from Alfa Aesar. 2-hydroxylethyl methacrylate (HEMA), ethyl α-bromoisobutyrate (EBiB) and ammonia were purchased from Merck. Copper chloride (CuCl), copper bromide (CuBr_2_), 2,2’-bipyridin and anhydrous methanol were purchased from Sigma Aldrich. Silver carbonate (Ag_2_CO_3_) was purchased from VWR. Diethyl ether and ethanol were purchased from Th. Geyer. The ATRP initiator 3-(trimethoxysilylpropyl)-2-bromo-2-methylpropionate was purchased from abcr and poly(dimethylsiloxane) (PDMS) was purchased from Dow Corning as Sylgard 184 Elastomer Kit. For the biological application the salt for PBS buffer, sodium chloride (NaCl), potassium chloride (KCl) and calcium chloride (CaCl_2_) were purchased from VWR. The disodium phosphate (Na_2_HPO_4_) was purchased from Sigma Aldrich and the potassium phosphate (KH_2_PO_4_) was purchased from Roth. The lectins GS-II and ECL were purchased from Vector Laboratories.

### 2.2. Analytics

Nuclear magnetic resonance (NMR) spectra were performed with a Unity INOVA 500 NB (Varian) 50-500 MHz using deuterium oxide (D_2_O, 99.9%) and deuterated chloroform (CDCl_3_, 99.9%) as solvent. For contact angle measurements, a water droplet (2 μL) was placed on the surface and the contact angles were analyzed by an OCA 15 dataphysics analyzer. X-ray photoelectron spectroscopy (XPS) measurements were performed with a Kratos Axis 165 with a monochromatic Al-source. Atomic force microscopy (AFM) was performed with a Dimension Icon microscope from Bruker Cooperation. The images were taken in tapping mode. The fluorescence microscopy measurements were performed on a Leica DMi8.

### 2.3. Production of Ring Resonators

The optical ring resonators were fabricated on 4-inch base wafers. They had a layer stack of 200 nm silicon nitride (Si_3_N_4_) grown by low-pressure chemical vapor deposition (LPCVD), 3.4 µm thermal silicon dioxide (SiO_2_) on 525 µm silicon (Si). A positive tone photoresist was used as the etch mask for the optical waveguides during the lithographic processes. They were etched 150 nm into the silicon nitride layer by reactive ion etching (RIE) and featured a width of 1.5 µm, ensuring single mode operation at a wavelength of 1550 nm. The photoresist was removed by rinsing in N-methyl-2-pyrrolidone (NMP). Finally, 200 nm SiO_2_ was deposited by plasma-enhanced chemical vapor deposition (PECVD) as a coating, which was locally removed in square windows around the ring resonators to allow for the interaction with the sample.

### 2.4. Synthesis Glycomonomer GlcNAcEMA

The glycomonomer GlcNAcEMA was synthesized using a modified literature procedure [11]. The first step was a Horton [19] reaction: Acetyl chloride (40.0 mL) was stirred with dried GlcNAc (20.0 g, 90.4 mmol) for 24 h. After purification and crystallization from diethyl ether, Ac-GlcNAc-Cl was obtained as off-white powder (23.8 g, 65.1 mmol, 72.0%) [19]. The NMR-spectrum of Ac-GlcNAc-Cl is provided in the Supplementary Materials.

^1^H NMR (CDCl_3_, 500 MHz): 6.19 (d, J = 3.66 Hz, 1H, α-H1); 6.17 (d, J = 3.66 Hz, 0.4H, β-H1); 5.85 (d, J = 8.55 Hz, 1H, NH); 5.32 (t, J = 9.52 1H, H3); 5.22 (m, 1H, H4); 4.53 (m, 1H, H2); 4.26 (m, 2H, H5/H6); 4.12 (m, 1H, H6); 2.10 (s, 3H, OCH3), 2.05 (s, 6H, 2xOCH3); 1.99 (s, 3H, NHCH3) ppm.

The second step was a Königs–Knorr reaction: A solution of HEMA (11.0 mL, 93.4 mmol) and Ag_2_CO_3_ (17.2 g, 62.3 mmol) were stirred in anhydrous dichloromethane with molecular sieve for 2 h. Ac-GlcNAc-Cl (22.0 g, 62.3 mmol) and AgClO_4_∙H2O (0.74 g, 3.11 mmol) was added to the solution and stirred for another 24 h. The solution was purified and crystallized to obtain Ac-GlcNAcEMA as an off-white powder (3.20 g, 6.96 mmol, 10.0%) [20,21], which was analyzed by NMR. The recorded ^1^H spectrum auf this compound can be found in the Supplementary Materials.

^1^H NMR (CDCl_3_, 500 MHz): 6.12 (s, 1H, C=CH2); 5.62 (d, J = 8.79 Hz, 1H, NH); 5.59 (t, J = 1.46 Hz, 1H, C=CH2); 5.28 (m, 1H, H3); 5.07 (t, J = 9.7 Hz, 1H, H4); 4.75 (d, J = 8.30 Hz, 1H, H1); 4.40 (m, 1H, CH2OCO); 4.24 (m, 2H, H5/H6); 4.12 (dd, J = 2.22, 14.65, 1H, H2); 4.03 (m, 1H, H6); 3.85 (m, 2H, OCH2); 3.71 (m, 1H, CH2OCO); 2.09 (s, 3H, OCH3), 2.02 (s, 6H, 2xOCH3); 1.91 (s, 3H, NHCH3) ppm.

The third step obtained deacetylated GlcNAcEMA: Ac-GlcNAcEMA (3.62 g, 7.88 mmol) was dissolved in anhydrous methanol, NaOCH_3_ (42.5 mg, 0.788 mmol) was added and the reaction mixture was stirred for 30 min. The reaction mixture was reduced and obtained by lyophilization as an off-white powder (0.170 g, 10.9 mmol, 95.0%) [11]. The NMR spectrum auf the final monomer GlcNAcEMA is shown in the Supplementary Material.

^1^H NMR (D2O, 500 MHz): 6.10 (s, 1H, C=CH2); 5.69 (s, 1H, C=CH2); 5.50 (d, J = 8.55 Hz, 1H, H1); 4.26 (m, 1H, H4); 4.06 (m, 1H, H3); 3.87 (m, 2H, CH2OCO); 3.65 (m, 3H, H5/H6a/b); 3.49 (m, 1H, H2); 3.39 (m, 2H, OCH2); 1.91 (s, 3H, OCH3); 1.88 (s, 3H, NHCH3) ppm.

### 2.5. Microcontact-Printing

Poly(dimethylsiloxane), or PDMS, was cut into small pieces and activated with air plasma (0.2 mbar, 5 min). The PDMS-stamps were inserted in a solution of ethanol (25.0 mL), ammonia (2.10 mL) and 3-(trimethoxysilylpropyl)-2-bromo-2-methylpropionate (250 μL) for 1 h. The stamps were dried in a nitrogen stream and incubated again for 3 min with a solution of ethanol (2.28 mL), ammonia (0.24 mL) and 3-(trimethoxysilylpropyl)-2-bromo-2-methylpropionate (120 μL). The stamps were dried and stamped on freshly with air plasma (0.2 mbar, 5 min) with activated ring resonators for 30 s, with a weight of 16.0 g. The ring resonators were rinsed with ethanol and water and dried in a nitrogen stream.

### 2.6. Synthesis of Glycopolymers on Ring Resonators

The glycopolymers were polymerized by surface-initiated atom transfer radical polymerization (SI-ATRP) on the ring resonators. A standard preparation was as follows: The initiator modified ring resonators were placed in a glass tube and flushed with nitrogen. The glycomonomer GlcNAcEMA (0.50 g, 1.56 mmol), CuBr_2_ (6.80 mg, 0.031 mmol), CuCl (12.0 mg, 0.125 mmol) and 2,2’-bipyridin (48.8 mg, 0.312 mmol) was dissolved in 15.0 mL methanol/water (1/1, v/v) and degassed with nitrogen. The solution was transferred to the tube containing the ring resonators. Free initiator EBiB (4.57 μL, 0.031 mmol) was added and the reaction mixture was stirred in an ice bath at 0 °C for 16 h. The ring resonators was rinsed with ethanol and water and dried in a nitrogen stream. The ring resonators were used for further measurements.

### 2.7. Binding Analysis Using Optical Ring-Resonators

For the binding analysis of lectins to glycopolymers the coated ring resonators were washed with buffer solution (PBS + 0.1 mM CaCl_2_). A droplet of a solution containing the buffer (PBS + 0.1 mM CaCl_2_, 1980 μL/1992 μL) and lectin (final concentration 20 μg/mL; GS II or ECL) was added on the ring resonator and the resonance wavelength shift was measured. Transverse-electrical polarized light from a Yenista TUNICS T110R tunable laser was coupled into the bus waveguide of the optical ring resonator by a first lensed optical fiber, collected by a second lensed optical fiber at the output of the sensor chip and detected in a Yenista CT400 optical component tester. A wavelength sweep between 1500 nm and 1550 nm was conducted every 3 s, allowing for the monitoring of resonance wavelength as a function of time.

## 3. Results and Discussion

**Figure 1:**
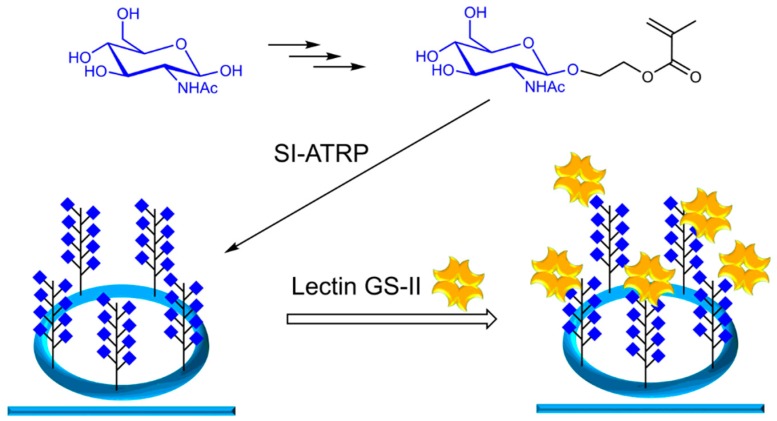
Sketch of the presented sensor system: Glycomonomers are synthesized and grafted from silicon nitride-based optical ring resonators via controlled radical polymerization. Lectin binding studies are performed on the surfaces.

We used polymerizable *N*-acetylglucosamine (GlcNAc)-derivatives for the surface grafted polymerization of glycopolymers via surface-initiated atom transfer radical polymerization (SI-ATRP). The sensors were analyzed after each step by contact angle measurements, atomic force microscopy (AFM) and X-ray photoelectron spectroscopy (XPS) to prove the successful functionalization of the silicon nitride surface. Binding of the lectin GS-II from *Griffonia simplicifolia*, which is selective for GlcNAc [22], and lectin ECL from *Erythrina cristagalli* [23], which binds terminal galactose and therefore acts as negative control, were analyzed by fluorescence microscopy in the first place and by utilizing the actual ring resonator coated with glycopolymers (Figure 1).

**Figure 2:**
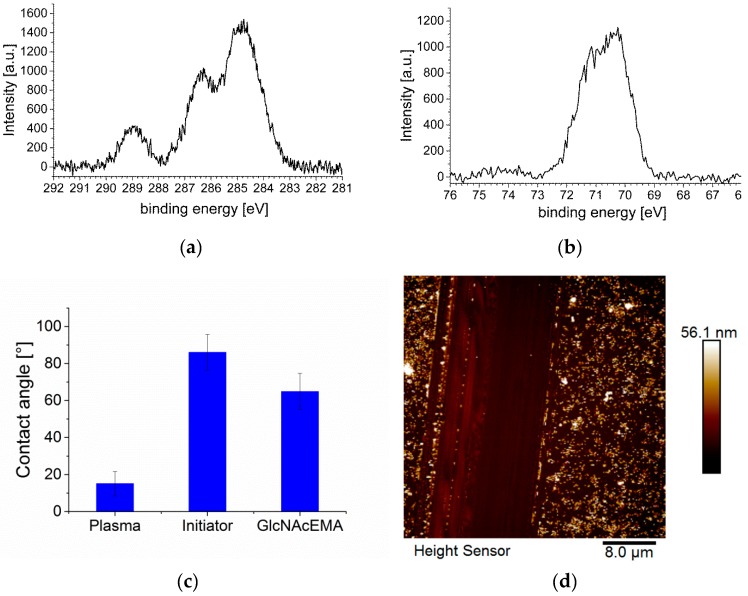

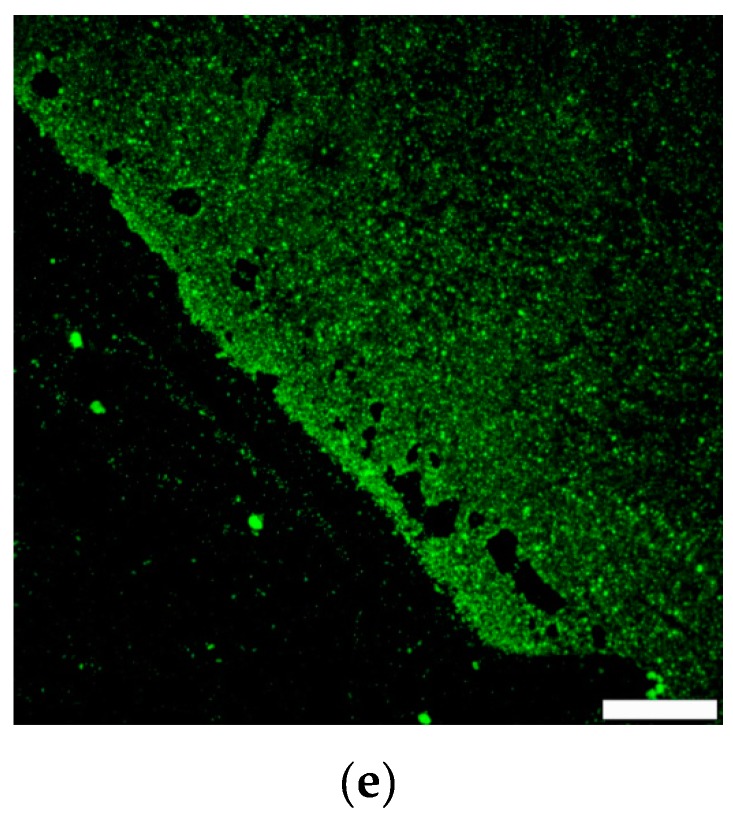
(**a**) and (**b**): X-ray photoelectron spectroscopy (XPS) data of the Si_3_N_4_ surface with the bound initiator, (**a**) is the spectrum of 1s carbon shell; (**b**) is the spectrum 3d bromide shell from which the electrons are released. (**c**) Contact angle measurement of Si_3_N_4_ surface, before modification, with bound initiator and after the polymerization with PGlcNAcEMA. (**d**) Atomic force microscopy (AFM) height image, left side untreated Si_3_N_4_ surface and right side coated with PGlcNAcEMA. (**e**) Fluorescent image of lectin binding to the glycopolymer PGlcNAcEMA, left side (black) untreated Si_3_N_4_ surface and right side coated with PGlcNAcEMA (green fluorescence signal).

First, GlcNAc based glycomonomers were synthesized. Although laborious, we choose the Horton reaction for protection and activation of the anomeric C-atom, as well as the Koenig–Knorr reaction as the needed amount of monomers was—due to the small footprint of the sensor—very low. The overall synthesis yield was about 10%. One actual key question was, whether the initiator used for functionalization of the silicon surfaces was also usable for silicon nitride surfaces. Thus, we applied short air–plasma treatment of silicon nitride surfaces to yield reactive hydroxyl groups on the surface, without altering the properties of the resonators beyond that. The efficient deposition of the initiator was proven by XPS, which clearly showed the presence of bromide, which was abundant in the initiator silane (Figure 2a,b). In the X-ray photoelectron spectroscopy (XPS) spectrum of the 1s carbon shell, we determined three signals. The first signal at 289 eV could be assigned to the ester/carbonyl group of the initiator. The second signal at 287 eV comprised the ester group and the third signal at 285 eV were carbon atoms of the C-C linkage. The XPS spectrum of the 3d bromide shell showed one significant signal for the bromide group in the initiator. The elemental composition additionally demonstrated that the initiator was attached to the Si_3_N_4_ surface: We recorded a high nitrogen (31.0%) and silicon (33.4%) content due the Si_3_N_4_ surface. We measured 9.7% carbon, 25.0% oxygen and 0.8% bromide on the surface. The concentration of 25.0% of oxygen was related to the generated hydroxide groups on the Si_3_N_4_ surface by plasma treatment. The elemental surface composition can be found in Table S1 in the Supplementary Material.

Additionally, the increase in hydrophobicity indicated successful deposition of the compound. For better analysis, we performed micro-contact printing (µCP) of the initiator to achieve an intrinsic negative control without polymer. For this we utilized poly(dimethylsiloxane) PDMS stamps. To stamp the initiator on the Si_3_N_4_ surface, the PDMS stamps were inserted and incubated with an initiator solution and then stamped on the surface with pressure. We varied the inserting time, the incubation time and the stamping time. The optimized processes, where we got a homogeneous initiator film on the surface, was the standard process as described in the experimental section, with an inserting time of 1 h, an incubation time of 3 min and a stamping time of 30 s.

After polymerization via SI-ATRP a thickness of the polymer layer of about 50 nm was estimated by AFM and a change in contact angle hinted for the production of glycopolymers (Figure 2c,d). Due to the fact that only a limited area was covered with polymer by printing the initiator layer, the thickness could be easily determined by AFM. Fluorescence microscopy using fluorescein-labeled GS-II showed only a signal when applied on initiator-printed surfaces (Figure 2d). Washing of the surfaces was obligatory to remove nonspecifically bound lectin from the non-coated parts. So far, the characterization proved a successful functionalization of silicon nitride surfaces with GlcNAc-based glycopolymers.

**Figure 3:**
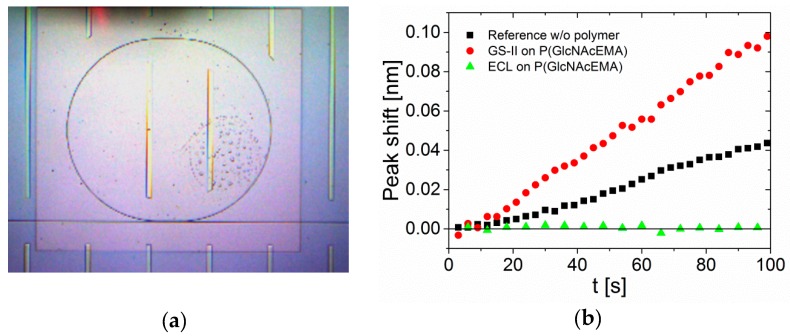
Microring resonator, (**a**) microscope top view of a ring resonator, (**b**) microring resonator measurements, wavelength shift as a function of time; (black) reference ring, binding of GS-II to uncoated surface; (red) binding of GS-II to ring resonator coated with PGlcNAcEMA; (green) binding of ECL to microring resonator coated with PGlcNAcEMA.

Next, we analyzed binding of lectins to the surface by spectroscopy using the actual optical ring resonator. The binding of molecules to the ring resonator surface induced an alteration of resonance and ultimately a small shift in wavelength, which could be measured. This wavelength shift is shown in Figure 3b over time, with a measure for molecule interactions or mass deposition on the surface.

This time, label-free lectins were used. To keep the system as simple and applicable as possible, we simply spotted a drop of buffer solution, containing 20 µg/mL lectin, onto the coated ring resonators and recorded the shift of wavelength (Figure 3).

We compared binding of GS-II to the GlcNAc-polymer surface, to a non-treated bare sensor. Clearly, GS-II showed more binding on the glycopolymer-treated surfaces. However, as expected, the nonspecific binding to the reference ring was rather strong. As the system should work without additional blocking or washing steps, this could be improved in the future by using glycopolymers that are not recognized by the analyzed lectin. It is notable that lectins in general show high unspecific adsorption to surfaces. To be able to differentiate between specific and unspecific binding, we used ECL as GlcNAc non-binder as reference on GlcNAc-glycopolymer presenting sensors. Interestingly, no signal at all was detected with ECL. The wavelength shift depended on the molecular weight of the binding compounds: Larger molecules exhibited a larger change in the refractive index of the chip surface. The molecular weight of the two binding proteins was different. ECL has a molecular weight of 54 kDa and GS-II of 113 kDa, which could lead to higher signals for GS-II. However, there was no signal change in the measurements with ECL at all, which led to the conclusion that there was indeed no interaction between ECL and the GlcNAc-presenting surface.

This hints not only the low unspecific adsorption of proteins onto the glycopolymers, but also the glycan-specific interaction of the recorded binding events. The maximum measurement time was 100 s, but even shorter times were sufficient for detecting clear differences in lectin binding. Increasing the measurement time beyond 100 s led to signal corruption, most probably due to evaporation of the buffer. The gathered data is shown in the Supplementary Material. Signal saturation was not observed during these measurement times as no complete coverage of all available binding sites took place within this time frame without a fluidic sample deployment. Under static conditions we assumed that the binding was rather slow and increased measurements times would lead to corrupted signals as stated above. However, as a drop-test approach was in focus, fast measurements were favored. The glycopolymer-coated optical ring resonator gave reasonable yes/no answers on lectin binding with a very simple measurement setup. We used lectin concentrations of 20 µg/mL and achieved very clear signals within 3 min run-time. The initial concentration may be reduced by approximately 10-fold and still yielded sufficient signals, compared to the reference with non-binding lectins after 100 s measurement time. This could enable analysis of lectin concentrations in the nanomolar range, which could be part of future work.

## 4. Conclusions

This simple approach, which avoided fluidical systems and the simple spotting of samples, together with the possibility of multiplexing and easy scale-up of production makes optical ring resonators and glycopolymers a very good pair, with regard to lectin-based diagnostics in the future. In general, kinetic analysis are also possible, but for this a fluidic system must be employed. For detecting the presence or absence of lectins or lectin-domain bearing proteins in a drop-test within very short (< 3 min) analysis times, this system seems to be an excellent choice. In the future, the system will be equipped with other glycan ligands and tested with lectins of medical interest.

## Supplementary Materials

The following are available online at https://www.mdpi.com/2079-6374/9/1/24/s1, Figure S1: ^1^H NMR spectrum of Ac-GlcNAcCl. Figure S2: ^1^H NMR spectrum of Ac-GlcNAcEMA. Figure S3: ^1^H NMR spectrum of GlcNAcEMA. Table S1: Elemental composition of the Si_3_N_4_ surface. Figure S4: Original measured binding data.
