# Supplementary material for: Rapid Drop-Test for Lectin Binding with Glycopolymer-Coated Optical Ring Resonators
Source: Biosensors (Basel). 2019 Feb 12;9(1):24. doi: 10.3390/bios9010024 (PMC6469017; doi:10.3390/bios9010024)
Supplement: Supplementary file 1 [file biosensors-09-00024-s001.pdf]

biosensors

Supporting Information

Rapid Drop-Test for Lectin Binding with Glycopolymer-Coated Optical Ring Resonators

Christine Schulte-Osseili [1], Moritz Kleinert [2], Norbert Keil [2] and Ruben R. Rosencrantz [1,*]

[1] Fraunhofer Institute for Applied Polymer Research IAP, Geiselbergstr. 69, 14476 Potsdam, Germany; christine.schulte-osseili@iap.fraunhofer.de
[2] Fraunhofer Institute for Telecommunications, Heinrich Hertz Institute, HHI, Einsteinufer 37, 10587 Berlin, Germany; moritz.kleinert@hhi.fraunhofer.de (M.K.); norbert.keil@hhi.fraunhofer.de (N.K.)
* Correspondence: ruben.rosencrantz@iap.fraunhofer.de, Tel.: +49-331-568-3203

Table of contents

Figure S1: ^{1}H NMR spectrum of Ac-GlcNAcCl, CDCl3 2

Figure S2: ^{1}H NMR spectrum of Ac-GlcNAcEMA, CDCl3 2

Figure S3: ^{1}H NMR spectrum of GlcNAcEMA, CDCl3 3

Table S1: Elemental composition of the Si3N4 surface after deposition of the initiator 3

Figure S4: Measured binding data 4

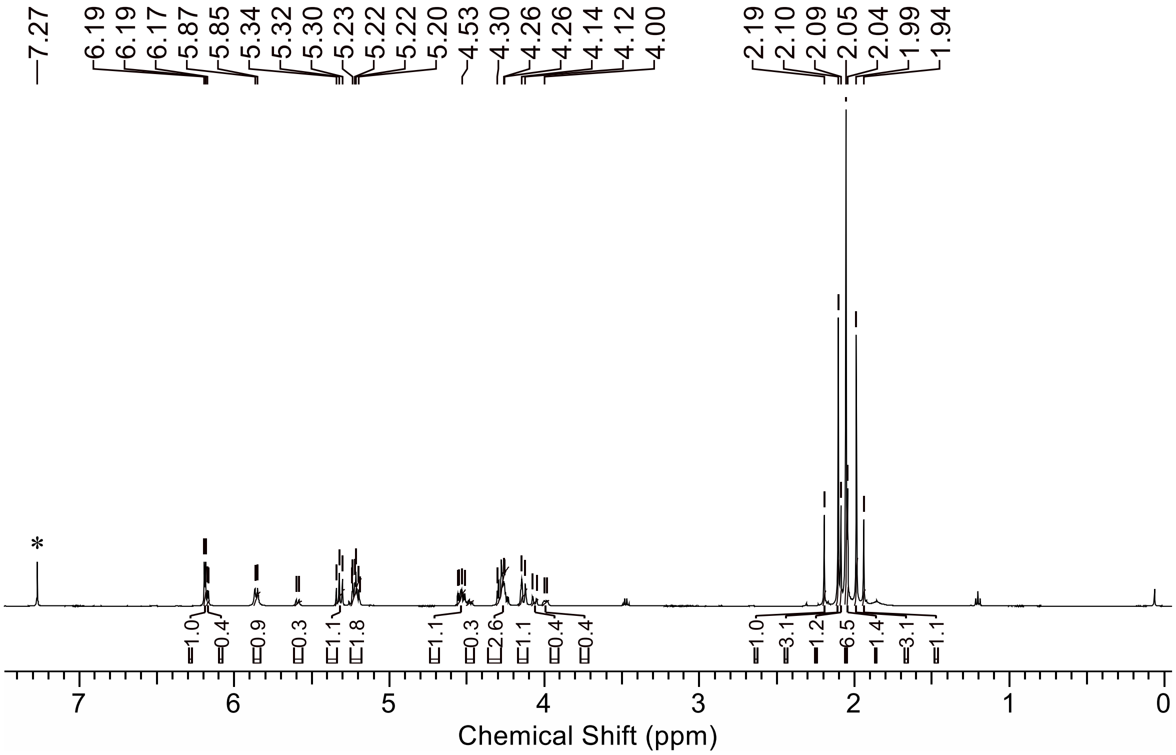

Figure S1: ^{1}H NMR spectrum of Ac-GlcNAcCl, CDCl3 (*).

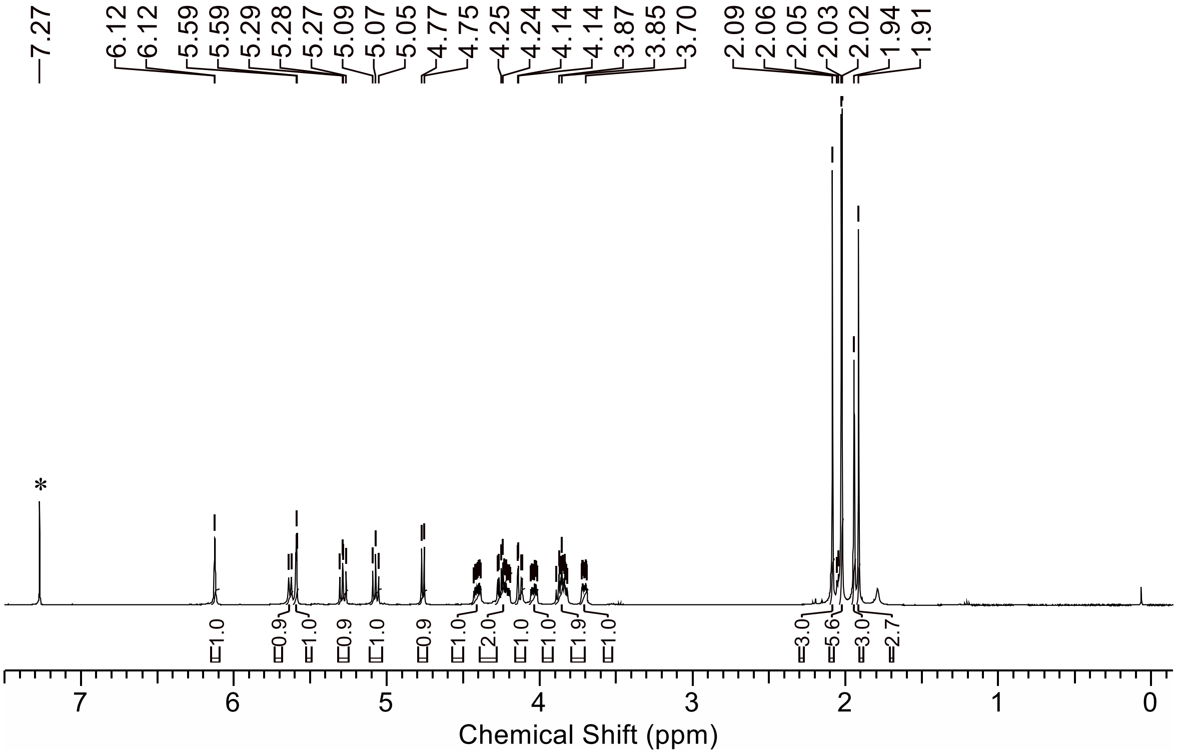

Figure S2: ^{1}H NMR spectrum of Ac-GlcNAcEMA, CDCl3 (*).

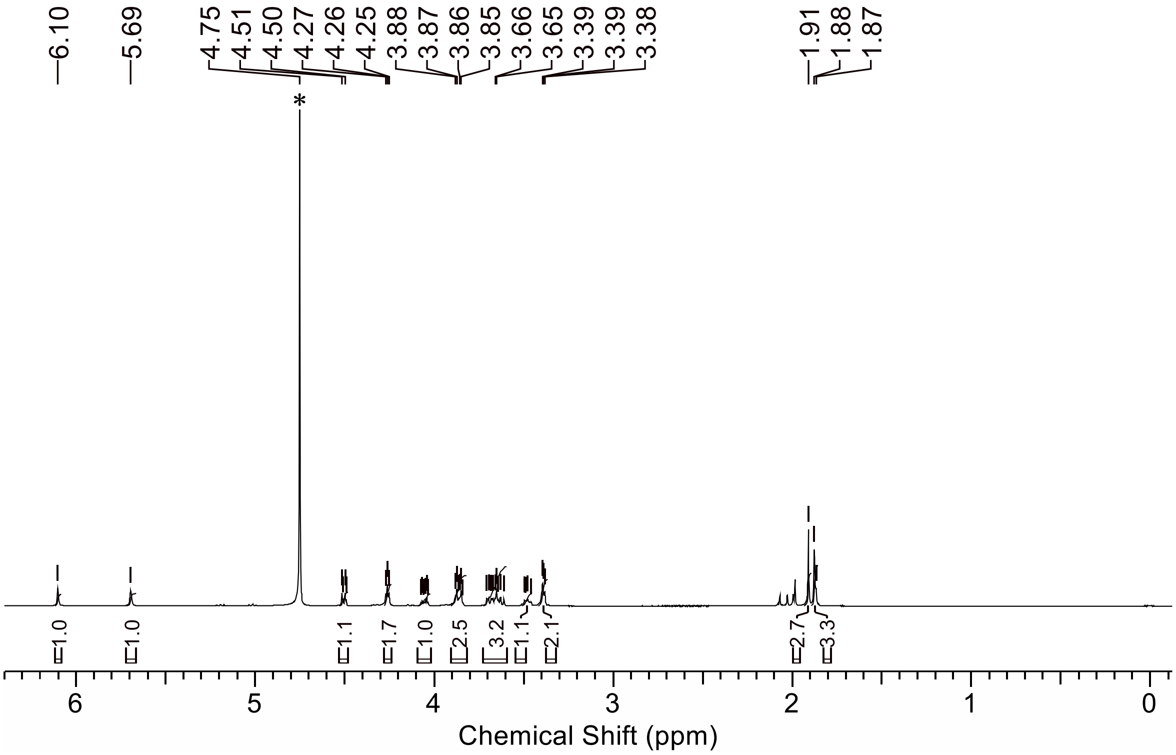

Figure S3: ^{1}H NMR spectrum of GlcNAcEMA, CDCl3 (*).

Table S1: Elemental composition of the Si3N4 surface after deposition of the initiator

	**Elemental concentration [%]**				
	C	N	O	Si	Br
Si_3N_4	9.7	31.0	25.0	33.4	0.8

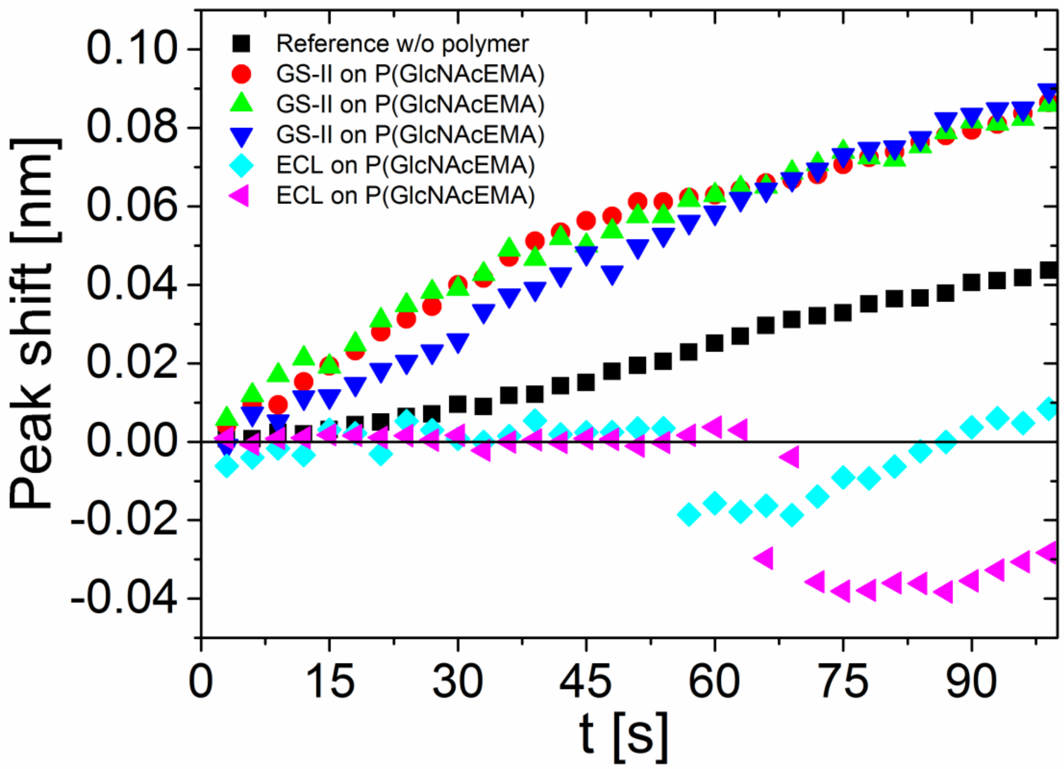

Figure S4: Measured binding data: Multiple measurements performed with GS-II on P(GlcNAcEMA) coated optical ring resonators and ECL as non-binder. Black data shows unspecific adhesion to the sensor surface without polymer coating. The measurements were performed on different chips.
